# Effect of 8-Week Consumption of a Dietary Pattern Based on Fruit, Avocado, Whole Grains, and Trout on Postprandial Inflammatory and Oxidative Stress Gene Expression in Obese People

**DOI:** 10.3390/nu15020306

**Published:** 2023-01-07

**Authors:** Diana María Muñoz-Pérez, Clara Helena González-Correa, Elcy Yaned Astudillo Muñoz, Maite Sánchez-Giraldo, Juan Carlos Carmona-Hernández, José López-Miranda, Antonio Camargo, Oriol Alberto Rangel-Zúñiga

**Affiliations:** 1 Grupo de Investigación Nutrición, Metabolismo y Seguridad Alimentaria, Departamento de Ciencias Básicas de Salud, Universidad de Caldas, Manizales 170004, Colombia; 2 Grupo de Investigación NutriOma, Facultad de Ciencias de la Salud, Universidad Libre Pereira, Pereira 660001, Colombia; 3 Grupo de Investigación Gerencia del Cuidado, Facultad de Ciencias de la Salud, Universidad Libre Pereira, Pereira 660001, Colombia; 4 Lipids and Atherosclerosis Unit, Department of Internal Medicine, Reina Sofia University Hospital, 14004 Córdoba, Spain; 5 Department of Medical and Surgical Sciences, University of Córdoba, 14004 Córdoba, Spain; 6 Maimonides Biomedical Research Institute of Cordoba (IMIBIC), 14004 Córdoba, Spain; 7 Grupo de Investigación Médica, Línea Metabolismo-Nutrición-Polifenoles (MeNutrO), Universidad de Manizales, Manizales 170004, Colombia; 8 CIBER Fisiopatología de la Obesidad y Nutrición (CIBEROBN), Instituto de Salud Carlos III, 28029 Madrid, Spain

**Keywords:** gene expression, healthy diet, inflammation, obesity, oxidative stress

## Abstract

Overweight and obesity constitute a major global public health problem. Healthy dietary patterns induce changes at the molecular level. Currently, there are no studies evaluating the effect of a diet based on fruit, avocado, whole grains, and trout (FAWGT diet) on the expression of obesity-related genes. This randomized controlled crossover study included 44 obese Colombians with BMI ≥30 kg/m^2^ who followed either a FAWGT diet or a usual diet (UD) characterized by a high intake of saturated fat and foods rich in processed carbohydrates. After 8 weeks of intervention, a postprandial expression study of inflammation and oxidative stress-related genes was carried out with a real-time PCR. The intervention with a FAWGT diet decreased the expression of inflammatory (*NFKB1*, *IL6*, *IL1β*) and oxidative stress (*NFE2L2*) genes compared with the intake of the UD. Finally, the postprandial expression of *NFkB1* was positively correlated with triglyceride levels after a dietary intervention with the FAWGT diet and the *IL1β* gene, and likewise with insulin levels after following the usual diet. The consumption of the FAWGT diet for 8 weeks reduced the inflammatory status; thus, it can be considered a valid alternative to other healthy diets, since it induces beneficial changes on the genes involved in inflammation and oxidative stress in obese people.

## 1. Introduction

The incidence of obesity in the world has tripled over the last 40 years, and overweight and obesity have now reached pandemic proportions and become a global health problem [1]. In 2016, over 1.9 billion adults were overweight, of whom over 650 million were obese [2]. Obesity is considered a starting point for the development of other diseases with high mortality rates, such as cardiovascular disease, type 2 diabetes mellitus, and some cancers [3]. For this reason, efforts are being made to understand the mechanisms underlying overweight and obesity. Knowledge of these mechanisms allows us to plan effective actions to modulate patients’ metabolism in order to keep their weight at healthy levels.

It has been proposed that a chronic low-grade inflammatory state, characterized by an increase in proinflammatory molecules, constitutes the link between obesity and chronic noncommunicable diseases such as obesity, and that the inflammation in obesity originates from dysfunctional adipose tissue due to the excess of nutrients. In this scenario, an infiltration of macrophages occurs, stimulating the expression of inflammatory genes such as the cytokines *IL1β* or *IL6* [4], which may be modulated by diet or specific nutrients, possibly through the activation of signaling pathways or by acting directly on transcription factors [3,5]. For example, an intervention comparing the consumption of a Mediterranean-rich diet with a Western dietary pattern that is rich in saturated fatty acids showed a decrease in the expression of the proinflammatory genes *p65* and *MCP1* [6]. Additionally, after a breakfast rich in SFA, Monfort et al. [7] found an increase in *IL1β* expression compared to a breakfast rich in polyunsaturated fatty acids and fiber.

One of the main actions adopted to manage obesity is the adherence to healthy lifestyle habits, such as physical activity and healthy diets. The Mediterranean diet and the Nordic diet have been accepted as healthy dietary patterns; in fact, previous studies have shown that adherence to these patterns improves fatty acid metabolism and decreases insulin resistance [8]. In addition, the consumption of these dietary patterns has been associated with a decrease in the activation of obesity-associated processes such as inflammation and oxidative stress [9,10,11].

Moreover, current lifestyle habits mean that people spend up to 16 h in the postprandial state, which can negatively affect their metabolism. In the human body, the postprandial state is characterized by changes in macro- and micronutrient concentrations, factors produced by the gut microbiota, and endocrine signals, among others, so it is a dynamic, complex state affecting almost all our organs and tissues. The postprandial response subsequently influences the metabolism and general health status [12,13].

A previous study by the research team showed that the intake of a dietary pattern based on fruits, avocado, whole grains, and trout typical of the Colombian coffee region improved lipemia and postprandial insulinemia in obese people, suggesting that this dietary pattern could be an alternative to other heart-healthy patterns such as the Mediterranean and Nordic diets in regions where basic foods from these two diets are scarce or unavailable [14]. However, there are currently no studies assessing the effect of following this dietary model on the regulation of genes involved in obesity-associated processes such as inflammation and oxidative stress.

Our aim was to evaluate the effect of 8 weeks of consumption of foods such as fruit, avocado, whole grains, and trout on postprandial inflammatory gene expression (*NFKB1, RELA, IKKA, MMP9, TNF, IL1β, IL6*) and oxidative stress (*NFE2L2*) in obese Colombians as alternative foods to those included in other healthy models such as the Mediterranean and Nordic diets.

## 2. Materials and Methods

### 2.1. Study Design and Participants

**Figure 1:**
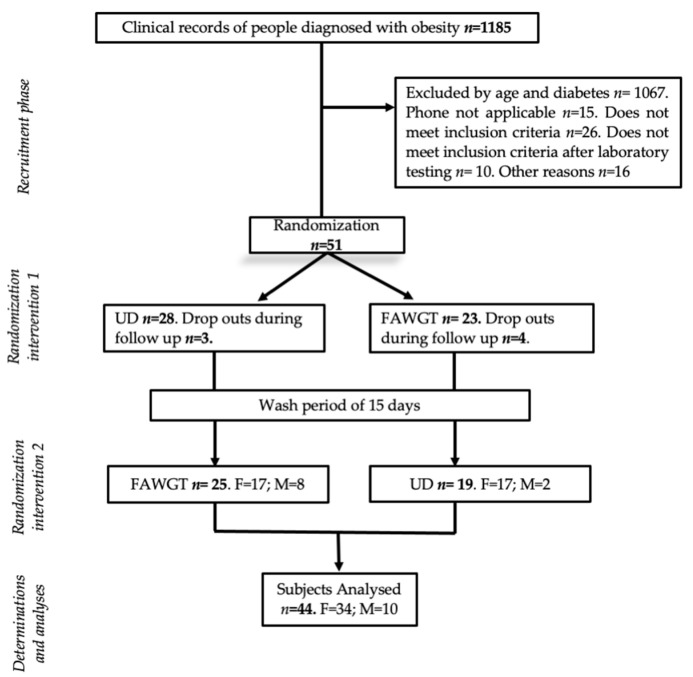
Flow chart of participants and study design. From 1185 medical records of patients diagnosed with obesity, 51 subjects were recruited, of whom 28 were randomized to follow the usual diet and 23 to the FAWGT diet. After 8 weeks of intervention, 7 subjects dropped out of the study and the remaining subjects changed their dietary pattern. Finally, 44 subjects completed the study. F, female; M, males; UD, usual diet; FAWGT, diet composed of fruit, avocado, whole grains, and trout.

The participants in the present study and its design were previously published [14]. In brief, the present study was a randomized controlled crossover study and the inclusion and exclusion criteria were previously published [14]. The sample size was calculated taking into account the main objective of the intervention, which was to analyze the postprandial change in triglycerides. Based on previous studies, 35 subjects were included with the aim of detecting a 15% change in postprandial triglycerides between the two dietary interventions, with a significance level of 0.05, a power of 80%, and a dropout rate of 10% [15]. After recruitment, 44 obese subjects with no diagnosed chronic diseases followed and completed the two dietary models for 8 weeks each, including 2 weeks of a washout period between diets (Figure 1). One of the diets was composed mainly of predominant foods of the Colombian coffee-growing zone (fruits, avocado, whole grain, and trout) (FAWGT diet) and the other diet consisted of foods that the participants consumed in their normal daily life, called the usual diet (UD). The subjects were instructed not to change their daily physical activity. All study participants provided their written informed consent, and ethics committees of the Universidad de Caldas and Clinica Comfamiliar approved the study protocol; additionally, this study is registered in ClinicalTrials.gov (NTC04920409).

### 2.2. Diet, Dietary Assessment, and Follow-Up Visits

The composition of the diets has been described in detail elsewhere [14,16]. The main differences between the diets at the nutrient level were the amount of dietary fiber and the quality of dietary fat, carbohydrates, and proteins. Both the FAWGT diet and the UD were isocaloric based on the evaluation of the habitual diet calculated from a 3-day food record before the beginning of the study. In the FAWGT diet, the main emphasis was on food items such as whole grains (*arepa* and rice), local fruits (e.g., *sweet granadilla/granadilla (Passiflora ligularis)*, *peach palm fruit/chontaduro (Bactris gasipaes)*, *cape gooseberry/uchuvas (Physalis peruviana)*, *star fruit/carambolo* (*Averrhoa carambola*), and *mango* (*Mangifera indica* L.)) and vegetables, rapeseed oil, avocado, and three fish (trout) meals per week. In the usual diet, participants were instructed to eat the foods consumed in their usual diet as part of their normal lifestyle. The usual diet was characterized by the consumption of refined cereals; foods rich in carbohydrates from bread, potato, plantain, and cassava; foods rich in saturated fat, with >50% fat especially in butter; <200 g/day of fruit and vegetables; <1 portion of any fish per week; consumption on demand of any type of meat per week; reduced consumption of legumes (<2 times per week); and an unrestricted consumption of sugary and carbonated beverages. The recommended foods for both diets are summarized in Table S1, where the macronutrient content was based on the Recommended Energy and Nutrient Intakes (RENIs) for the Colombian population [17]. The washout period consisted of a return to the patients’ usual diet characterized by a low consumption of fruit and vegetables, no fish, and whole grains.

The participants’ dietary follow-up protocol consisted of three 24 h reminders (two nonconsecutive during the week and one at the weekend). The recordings were carried out at the beginning of the dietary intervention (week 0), in the middle (week 4), and at the end (week 8) [14,16,18].

### 2.3. Postprandial Study

The present work lies within the framework of the postintervention phase of the study (Figure S1) after 8 weeks of dietary intervention during the postprandial state. Analyses were carried out between hour 0 and hour 4 of the postprandial state to assess the postprandial response after 8 weeks of intervention. The participants consumed breakfast based on the same composition of the diet into which they had been randomized for the dietary intervention period. The composition of the breakfasts for the postprandial study is shown in Table S2. During the postprandial period, participants could only drink water. The composition of the breakfasts was previously published [14], and in the FAWGT diet, it was based on the consumption of *arepa* prepared with whole grains, oats, typical fruits of the Colombian coffee region, and yogurt. The breakfast of the usual diet was based on the consumption of eggs, butter, whole milk, *arepa* prepared with refined flour, coffee with sugar, and the traditional *buñuelo* (fried food made of flour and cheese).

### 2.4. Biochemical Measurements of Metabolic Parameters

EDTA blood samples were collected from the participating patients after 12 h of fasting. In the present study, samples were used after 8 weeks of dietary intervention at time 0 and 4 h of the postprandial state (Figure S1). Clinical parameters associated with lipid metabolism, glucose homeostasis, and inflammation, which have been previously published, were measured from the samples obtained.

### 2.5. Peripheral Blood Mononuclear Cells (PBMC) Isolation

Venous blood samples were collected after 8 weeks of dietary intervention in tubes containing 1 g EDTA/L after 12 h of fasting at 0 h and 4 h after ingestion of the breakfast. The isolation of mononuclear cells was carried out with the Ficoll gradient and the cells obtained were stored at −80 °C in RNAlater (Invitrogen RNAlater™ Stabilization Solution, Invitrogen, Waltham, MA, USA) until use [19].

### 2.6. Total RNA Isolation and cDNA Generation

The RNA was isolated from PBMCs using the kit Direct-zol RNA Miniprep Plus according to the manufacturer’s instructions (Zymo Research, Irvin, CA, USA). The samples obtained were treated with DNAase I (AMPD-1 Kit, Sigma Aldrich, St. Louis, MO, USA), and then cDNA was obtained using the High-Capacity cDNA Synthesis Kit (Applied Biosystems, Carlsbad, CA, USA) for gene expression studies.

### 2.7. qRT-PCR Analysis of Gene Expression

A gene expression analysis was carried out via real-time PCR using the OpenArray platform according to the manufacturer’s instructions (Thermofisher Scientific, Waltham, MA, USA). The primers used for the genes studied were obtained from the Thermofisher Scientific website (https://www.thermofisher.com/es/es/home/life-science/pcr/real-time-pcr/real-time-pcr-assays/taqman-gene-expression.html. Accessed on 20 March 2021). The genes involved in the inflammatory and oxidative stress pathways were included in the OpenArray panel: *NFKB1, RELA, IKKA, MMP9, TNF, IL1β, IL6,* and *NFE2L2*. The gene expression was calculated with *HPR1*, *B2M,* and *GAPDH* as housekeeping genes, according to the Bestkeeper algorithm [20], and expressed as a relative expression using the equation:

relative expression = 2 − (Ct target gene − Ct bestkeeper).

The data set was analyzed using OpenArray^®^ Real-Time qPCR Analysis Software (Applied Biosystems, Carlsbad, CA, USA).

### 2.8. Statistical Analysis

Statistical analyses were carried out using SPSS 20 software. The values represent the mean and standard error. A comparison between the baseline characteristics was carried out using a one-way ANOVA. A comparison of the effects between the 0 h and 4 h postprandial status after 8 weeks of dietary intervention was performed using a repeated measures analysis. In the latter, we evaluated the overall effect of the dietary intervention (global ANOVA and *p* for diet), the effect of time postprandially (*p* for time), and the diet–time interaction (diet vs. time). For multiple comparisons, we used Sidak’s test. *p*-values < 0.05 were considered statistically significant. Finally, a correlation analysis was performed between the expression of inflammatory genes and the clinical and biochemical parameters of the participants using a Pearson bivariate correlation analysis with SPSS 20 for Windows software (SPSS Inc., Chicago, IL, USA), for which *p* < 0.05 was considered significant. The analysis was performed with the expression values of inflammatory genes and the values of clinical and biochemical parameters taken independently at times 0 and 4 h of the postprandial state after 8 weeks of dietary intervention for both diets—the FAWGT and usual diet.

## 3. Results

### 3.1. Effect of the Dietary Intervention on Clinical Variables of the Subjects Included in The Study

The baseline characteristics of the participants were previously published [14] (Table S3). Regarding the dietary intervention, we observed a lower weight and BMI (both *p* < 0.001) after the consumption of the FAWGT diet compared with the UD (Table 1). In contrast, no significant postprandial differences were observed after the two intervention periods in the other clinical parameters analyzed (Table S4).

### 3.2. Effect of the Dietary Intervention on the Postprandial Expression of the Inflammatory-Related Genes

**Figure 2:**
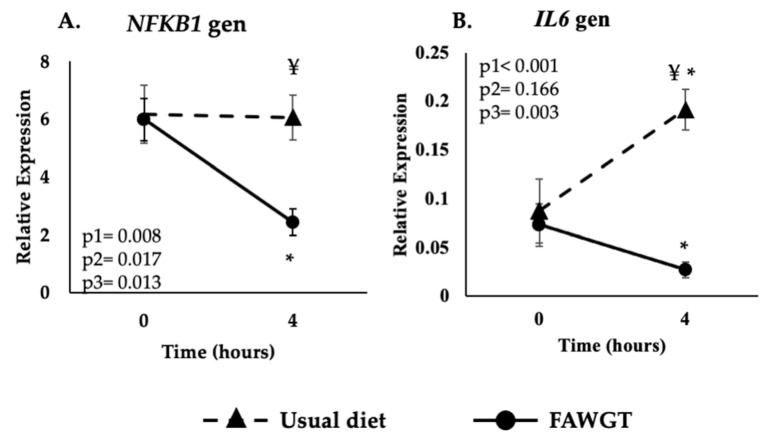
Postprandial expression of the inflammatory-related *NFKB1* (**A**) and *IL6* (**B**) genes after 8 weeks of dietary intervention with a usual diet and the diet consisting of fruit, avocado, whole grains, and trout predominant in the Colombia coffee region (FAWGT). Values are shown as mean ± S.E.M of the relative expression. The analyses correspond to ANOVA for repeated measures where p1: diet influence; p2: time, the kinetics of the postprandial response; and p3: diet–time interaction (diet vs. time). For multiple comparisons, we used the Sidak test. * *p* < 0.05 4 h vs. fasting state, ¥ *p* < 0.05 between diets at the same time.

The intake of the FAWGT diet for 8 weeks decreased the postprandial expression (4 h after meal intake) of the *NFKB1* gene compared to the fasting state (*p* < 0.001). In addition, the postprandial expression of the *NFKB1* gene was lower after the FAWGT diet than after the UD diet (*p* < 0.001) (Figure 2A).

Moreover, the intake of the FAWGT diet for 8 weeks decreased the postprandial expression of the *IL6* gene compared to the fasting state (*p* = 0.013) (Figure 2B). In contrast, the intake of the UD diet for 8 weeks increased the postprandial expression of the *IL6* gene compared to the fasting state (*p* = 0.027). In addition, the postprandial expression of the *IL6* gene was lower after the FAWGT diet than after the UD diet (*p* < 0.001) (Figure 2B).

**Figure 3:**
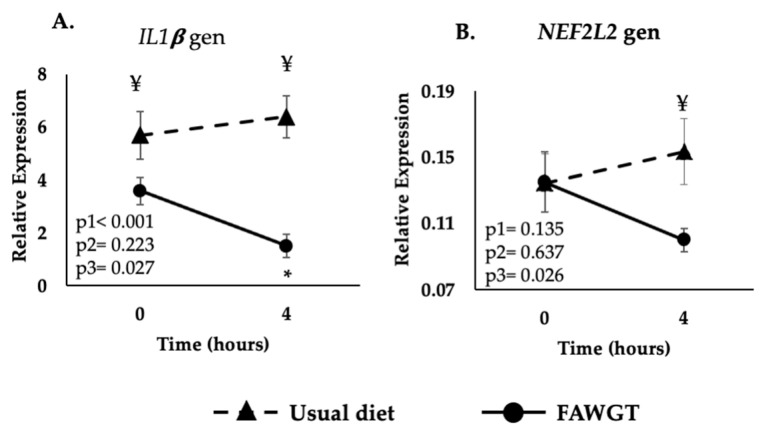
Postprandial expression of the inflammatory-related *IL1β* (**A**) and oxidative-stress-related *NFE2L2* (**B**) gene after 8 weeks of dietary intervention with a usual diet and the diet consisting of fruit, avocado, whole grains, and trout predominant in the Colombia coffee region (FAWGT). Values are shown as mean ± S.E.M of the relative expression. The analyses correspond to ANOVA for repeated measures where p1: diet influence; p2: time, the kinetics of the postprandial response and p3: diet–time interaction (diet vs. time). For multiple comparisons, we used the Sidak test. * *p* < 0.05 4 h vs. fasting state, ¥ *p* < 0.05 between diets at the same time.

The consumption of the FAGWT diet decreased the *IL1β* postprandial gene expression compared to the fasting state (*p* < 0.001). The *IL1β* gene expression was higher after the UD diet at both the fasting (*p* = 0.039) and postprandial state (*p* < 0.001) than after the consumption of the FAGWT diet (Figure 3A). In the other genes, no significant differences were found between the two diets.

### 3.3. Effect of the Dietary Intervention on the Postprandial Expression of the Oxidative Stress Gen

Finally, *NFE2L2* postprandial gene expression was higher after the consumption of the UD than after the FAWGT diet (*p* = 0.008) (Figure 3B).

### 3.4. Correlation Analysis between the Gene Expression of the Inflammatory and Oxidative Stress-Related Genes and Biochemical Parameters

**Figure 4:**
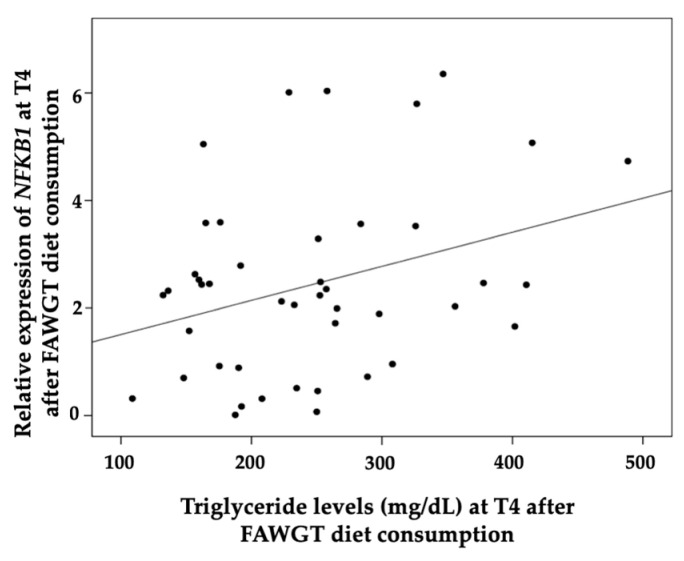
Correlation analysis between the gene expression of the inflammatory-related *NFKB1* and triglyceride levels in the postprandial state at 4 h after 8 weeks of dietary intervention with a diet consisting of fruit, avocado, whole grains, and trout predominant in the Colombia coffee region (FAWGT). The correlation was evaluated with a Pearson bivariate correlation analysis using SPSS 20 for Windows software (SPSS Inc., Chicago, IL, USA). *p* < 0.05 was considered to be significant.

**Figure 5:**
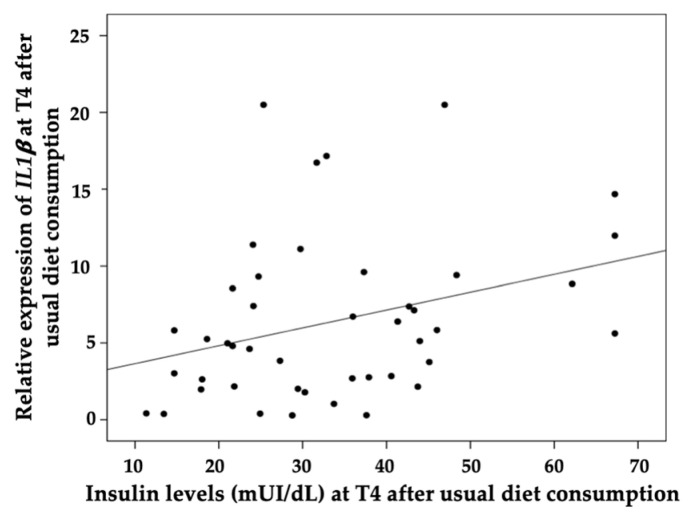
Correlation analysis between the gene expression of the inflammatory-related *IL1β* and the insulin levels in the postprandial state at 4 h after 8 weeks of intake of a usual diet. Correlation was evaluated with a Pearson bivariate correlation analysis using SPSS 20 for Windows software (SPSS Inc., Chicago, IL, USA). *p* < 0.05 was considered to be significant.

We observed a relationship between *NFKB1* gene expression and triglyceride levels 4 h after meal intake (r^2^ = 0.3241 and *p* = 0.032) after the consumption of the FAGWT diet (Figure 4). Moreover, the relative expression of *IL1β* was directly related to insulin levels at 4 h after the meal (r^2^ = 0.3168 and *p* = 0.0361) with the UD (Figure 5).

## 4. Discussion

Overweight and obesity currently constitute a major global public health problem, which is closely linked to the development of type 2 diabetes mellitus and cardiovascular disease. Previous studies have demonstrated the beneficial effect of healthy diets (Mediterranean and Nordic) on the regulation of genes associated with the development of these diseases. However, it is not possible to adhere to these dietary patterns in all regions of the world due to the cost and availability of foods. We aimed to evaluate the effect of the 8-week consumption of foods such as fruit, avocado, whole grains, and trout on postprandial inflammatory and oxidative stress gene expression in obese Colombians as alternative foods to those included in other healthy dietary models.

In a previous study, we described, in the same population that we had previously found, in the postprandial state, an increase in postprandial triglycerides, VLDL-c, and insulin levels after ingestion of the two diets; however, this increase was greater in the usual diet than in the diet based on fruit, avocado, whole grains, and trout (FAWGT diet) [14].

Here, our results show that in obese people, the intake for 8 weeks of the FAWGT diet decreases the postprandial expression of inflammatory (*NFKB1*, *IL6*, *IL1β*) and oxidative stress (*NFE2L2*) genes when compared with the intake of the usual diet. Finally, the postprandial expression of the *NFkB1* and *IL1β* genes correlated positively with triglyceride and insulin levels after the dietary intervention with the FAWGT diet and UD, respectively.

Obesity is the result of a complex interaction between genetic, metabolic, and environmental factors including dietary habits [21]. It has now reached pandemic proportions with an increase from 108 million people in 1982 to 422 million in 2014 [22]. If this trend continues, by 2030, more than 57.8% (3.3 billion people) of the world’s adult population will be overweight or obese [23]. Obesity has been associated with chronic low-grade inflammation and oxidative stress [24] characterized by the production of adipokines, proinflammatory cytokines, and reactive oxygen species, which are involved in the development of chronic noncommunicable diseases [1,25]. Moreover, the lifestyle of current society leads people to spend most of the day in a postprandial state. Staying in this state for more than 16 h leads to low-grade chronic inflammation, which is associated with the development of diseases [26].

Our nutrigenomic approach is based on the fact that nutrients and bioactive dietary compounds can modify gene expression. The discovery of these gene–nutrient interactions will help the use of customized diets. In line with this, this knowledge may allow for the design of dietary strategies focused on the reduction in the expression of inflammatory or oxidative stress genes, which is especially important in the case of noncommunicable diseases such as obesity, which are currently considered an important world public health problem [27].

In fact, previous studies have shown the effect of diet or its components on the molecular mechanisms associated with inflammatory activity and oxidative stress [28]. One study showed that the use of a Mediterranean diet model induced the downregulation of proinflammatory and endoplasmic reticulum stress genes, even after coenzyme Q10 supplementation, versus a diet model rich in saturated fats [29]. Additionally, the Nordic diet showed that this model reduced subcutaneous adipose tissue and inflammatory genes such as *IRF1*, *CD67*, *IL-32*, and *IL6R* compared to a control diet [30].

In the present study, the decrease in the postprandial expression of the proinflammatory genes (*NFKB1*, *IL6*, and *IL1β*) could have been due to the fact that the *NFKB1* gene encodes for the p50 protein, a subunit of the transcription factor NF-kB, which activates the transcription of proinflammatory genes (*TNF-α*, *IL1β*, *IL6*, among others), which regulate the inflammatory response [31]. The decreased postprandial expression of *IL6* and *IL1β* after 8 weeks of dietary intervention could be associated with the lower activation of the NF-kB transcription factor signaling pathway induced by the intake of healthy fatty acids from trout and avocado (omega 3, MUFA, and PUFA), which also improves postprandial triglyceride levels and glucose homeostasis [14,32,33].

In this context, a previous study showed that the consumption of a healthy Nordic dietary model induced the postprandial downregulation of inflammatory genes such as *TLR4, IL18*, and *CD36* and upregulated the expression of *PPARD* compared to a control diet in a population with metabolic syndrome [10], demonstrating, as in our study, that the consumption of healthy dietary patterns beneficially impacts the gene expression profile.

In line with the above, our observations may be explained by the composition of the FAWGT diet, characterized by the consumption of trout containing 1.4 g omega-3 (280 mg EPA and 160 mg DHA per 100 g) in addition to other nutrients such as selenium and vitamin D, among others [34]. Avocado also contains monounsaturated fatty acids, fiber, and phytonutrients, and both omega-3 and monounsaturated acids have been shown to regulate inflammatory markers. Thus, omega-3 fatty acids may act as ligands of the peroxisome-activated receptor (PPARγ ), a transcription factor that activates the expression of related genes with the inflammatory cascade [35,36,37]. Docosahexaenoic acid (DHA) inhibits the synergistic effect between palmitic acid and lipopolysaccharide (LPS) on the expression of proinflammatory genes via NF-kB [38]. Additionally, in vitro studies showed that avocado consumption decreased the concentration of proinflammatory molecules such as IL1β, IL6, and TNF-α through the modulation of the NF-kB factor [39]. Thus, these characteristics of the FAWGT diet account for a reduction in the inflammatory state, as suggested by the expression of the genes shown in this work.

In addition, the FAWGT diet also involves the consumption of typical fruit from the Colombian coffee region (e.g., *granadilla, chontaduro, uchuvas, carambolo,* and *mango*), which are rich in vitamins (C and D) and phenolic compounds with antioxidant power (β-carotenes and tocopherol) [40]. A previous in vitro study showed that in mononuclear cells, a multivitamin complex rich in b-carotenes, vitamin C, and tocopherol had an antioxidant and anti-inflammatory effect in patients with type 1 diabetes and decreased the production of proinflammatory cytokines IL6 and TNF-α and increased IL4 [41]. Moreover, the daily intake of 400 g/d of whole fruit (banana, tangerine, apple, strawberry, orange, peach, grape, and mango) for 14 days in young Colombians between 18 and 30 years old induced a reduction in the mRNA expression of *IL6R* and *RELA* genes and an increase in HDL cholesterol levels compared to a control group [42]. Another study demonstrated that the intake of *mango* tea had therapeutic potential in treating obesity and related diseases by regulating the expression of transcriptional factors and enzymes associated with adipogenesis [43].

The transcription factor NRF2 (encoded by the *NFE2L2* gene) regulates genes related to oxidative stress, which is directly associated with the inflammatory response [44]. In line with this, the current work showed a postprandial reduction in *NFE2L2* gene expression by the FAWGT diet, while the consumption of the UD had the opposite effect. These findings suggest that the effect of diet on the downregulation of inflammation, and therefore oxidative-stress-related genes, is due to a synergy between the main foods that constitute the dietary model, including the healthy fatty acids of animal and vegetable origin and local fruit, among others.

One limitation of this work is that the aim was to examine the effects of the FAWGT diet on the expression of some proinflammatory genes in peripheral blood mononuclear cells; however, the protein levels were not determined, which prevented us from relating gene expression with protein levels. Another limitation may be the small sample size; however, the study design allowed us to detect the net effect of the intervention with small working groups. The duration of the intervention period might be a limitation, and additional diet effects could be detected in a longer intervention. This should be taken into account for the extrapolation of the results, suggesting that further studies are needed to confirm our findings. On the other hand, one of the strong points of our study was the design; a randomized crossover study reduces interindividual variation, which allowed us to obtain reliable scientific evidence.

## 5. Conclusions

In conclusion, our results suggest that the consumption of a diet based on fruit, avocado, whole grains, and trout for 8 weeks reduces the postprandial inflammatory state, and can therefore be considered a valid alternative to other heart-healthy diets, since it improves the molecular regulation of the genes involved in the immune response and oxidative stress in obese subjects and demonstrates a beneficial effect at the postprandial state similar to other healthy diets.

## Figures and Tables

**Table 1:** Characteristics of subjects included in the study in fasting, after and before the dietary intervention.

	FAWGT		UD				
Parameter	Baseline	8 weeks	Baseline	8 weeks	*p* time	*p* diet	*p* Diet vs. Time
Weight (kg)	88.5 ± 2.0	87.0 ± 2.0 ^(a)^	88.3 ± 2.1	88.4 ± 2.1 ^(b)^	<0.001	0.007	<0.001
BMI (kg/m^2^)	35.7 ± 0.6	35.1 ± 0.7 ^(a)^	35.5 ± 0.6	35.6 ± 0.7 ^(b)^	0.001	0.015	<0.001
Fat (%)	42.7 ± 0.5	41.9 ± 0.6	42.4 ± 0.7	42.1 ± 0.6	0.006	0.748	0.285
Waist–hip ratio	0.90 ± 0.02	0.90 ± 0.01	0.91 ± 0.01	0.92 ± 0.01	0.488	0.017	0.764
Systolic blood (mmHg)	122.1 ± 1.8	119.8 ± 1.6	120.15 ± 1.9	120.0 ± 1.9	0.267	0.539	0.364
Diastolic blood (mmHg)	78.6 ± 1.4	77.6 ± 1.2	80.1 ± 1.2	77.6 ± 1.3	0.850	0.450	0.313
Glucose (mg/dL)	94.3 ± 1.6	94.4 ± 2.0	95.9 ± 1.7	96.0 ± 2.0	0.923	0.079	0.935
Insulin (mUI/mL)	22.5 ± 1.9	21.6 ± 1.4	20.16 ± 1.2	24.3 ± 1.8 ^(a,b)^	0.870	0.399	0.016
Total Cholesterol (mg/dL)	201.3 ± 5.5	201.3 ± 5.6	202.0 ± 5.3	200.4 ± 5.4	0.764	0.962	0.765
HDL-c (mg/dL)	40.1 ± 1.5	41.0 ± 1.4	43.4 ± 1.6 ^(b)^	41.6 ± 1.6 ^(a)^	0.494	0.005	0.039
Non-c HDL-c (mg/dL)	161.1 ± 5.8	160.3 ± 5.5	159.3 ± 5.5	159.0 ± 5.6	0.840	0.525	0.936
LDL-c (mg/dL)	118.4 ± 5.4	124.3 ± 5.3	118.0 ± 5.3	118.8 ± 4.8	0.212	0.303	0.439
TG (mg/dL)	198.2 ± 13.4	182.7 ± 10.1	191.2 ± 13	200.9 ± 14.3	0.726	0.444	0.100
CRP (mg/L)	4.35 ± 0.4	5.0 ± 0.5	4.6 ± 0.4	5.2 ± 0.4	0.002	0.326	0.898

FAWGT, diet rich in fruit, avocado, whole grains, and trout. UD, usual diet. Values represent the mean ± standard error. The analyses correspond to ANOVA for repeated measures, where *p* time is the kinetics of the dietary intervention response; *p* diet is the influence of diet; and *p* Diet vs. Time is the interaction of the two factors. When post hoc tests were pertinent, we used multiple comparisons with Sidak correction. (a) *p* < 0.05 by comparison to baseline values in the diet. (b) *p* < 0.05 between diets at the same time.

## Data Availability

The data presented in this study are available on request from the corresponding author C.H.G.-C. and the principal researchers of the project D.M.M.-P. and C.H.G.-C.

## Supplementary Materials

The following supporting information can be downloaded at https://www.mdpi.com/article/10.3390/nu15020306/s1: Figure S1: Study design; Table S1: Recommended foods in the study; Table S2: Composition and caloric distribution of the breakfasts used in the postprandial study; Table S3: Baseline characteristics of the patients included in the study before any dietary intervention; Table S4: Postprandial changes in the biochemical parameters after 8 weeks of dietary intervention.
