# Supplementary material for: Effect of 8-Week Consumption of a Dietary Pattern Based on Fruit, Avocado, Whole Grains, and Trout on Postprandial Inflammatory and Oxidative Stress Gene Expression in Obese People
Source: Nutrients. 2023 Jan 7;15(2):306. doi: 10.3390/nu15020306 (PMC9861897; doi:10.3390/nu15020306)
Supplement: Supplementary file 1 [file nutrients-15-00306-s001.zip › Supplementary Figures.pptx]

## Slide 1
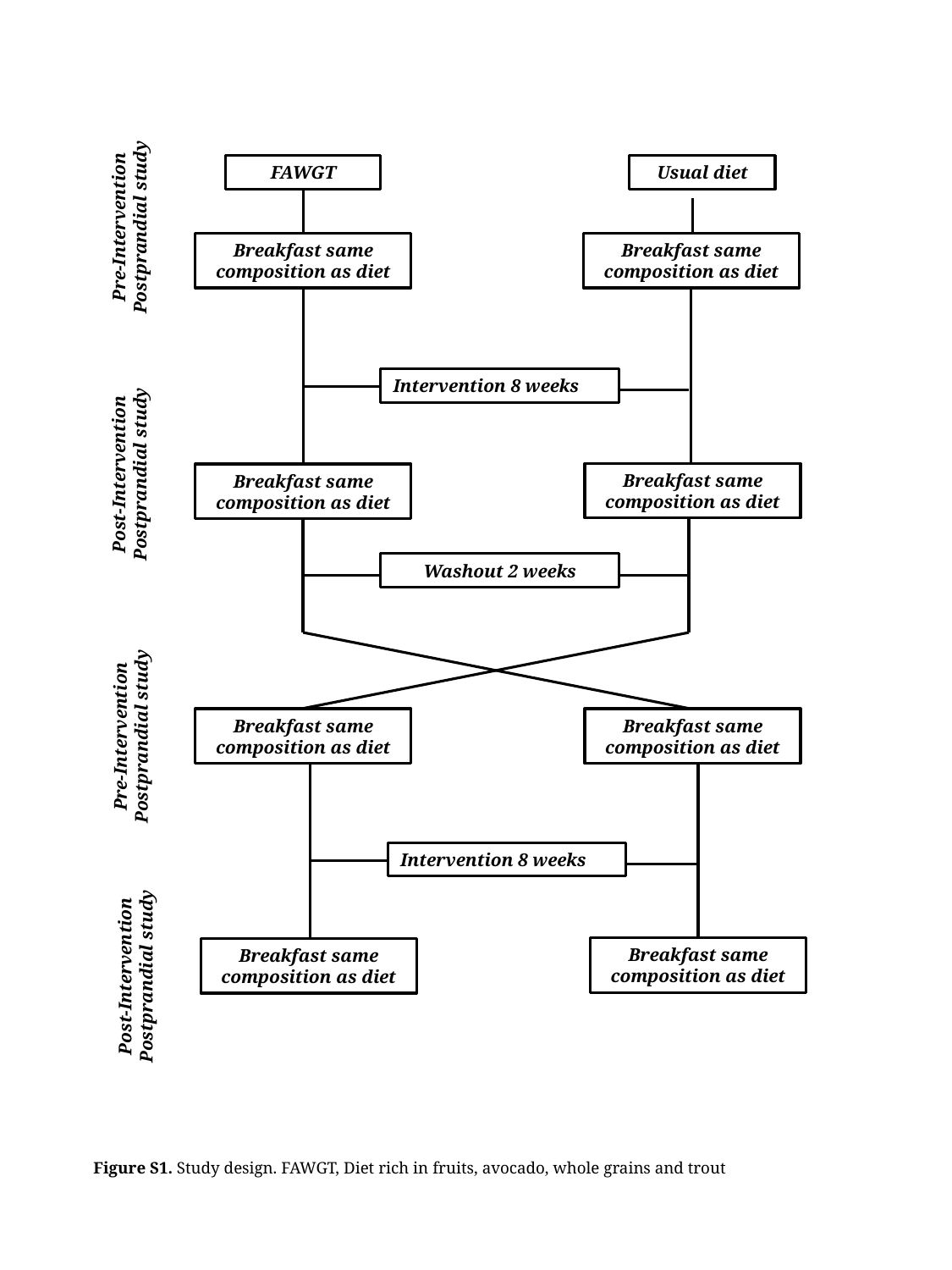

FAWGT
Usual diet
Pre-Intervention Postprandial study
Breakfast same composition as diet
Breakfast same composition as diet
Intervention 8 weeks
Post-Intervention Postprandial study
Breakfast same composition as diet
Breakfast same composition as diet
Washout 2 weeks
Breakfast same composition as diet
Breakfast same composition as diet
Pre-Intervention Postprandial study
Intervention 8 weeks
Breakfast same composition as diet
Breakfast same composition as diet
Post-Intervention Postprandial study
Figure S1. Study design. FAWGT, Diet rich in fruits, avocado, whole grains and trout
