# Supplementary material for: Effect of 8-Week Consumption of a Dietary Pattern Based on Fruit, Avocado, Whole Grains, and Trout on Postprandial Inflammatory and Oxidative Stress Gene Expression in Obese People
Source: Nutrients. 2023 Jan 7;15(2):306. doi: 10.3390/nu15020306 (PMC9861897; doi:10.3390/nu15020306)
Supplement: Supplementary file 1 [file nutrients-15-00306-s001.zip › Supplementary Tables.pdf]

Table S1. Recommended foods in the study.		
Food Group	*FAWGT*	*UD*
Cereals	Replacement of 50% of refined cereals by whole grains (rice, typical arepa and oats): rice of 80 grs, 1 arepa of 56 grs and oat of 24 grs per serving	Refined
Fat source	Canola, sunflower or soybean oil 5 grs per serving. Almonds 9 grs, nuts 8 grs and peanuts 10 grs per serving. Avocado 30 grs per serving.	Butter > 50% saturated fat
Fruits and Vegetables	Fruits 400 grs per serving (granadilla, uchuvas, orange or tangerine, guayaba, mango, apple) and vegetables 200 grs per serving (citron, spinach, beans, peas, carrots, tomatoes, onions, garlic). Typical chontaduro 27 grs, granadilla 100 grs, orange 147 grs per serving. Mango 112 grs, guayaba criolla 105 grs per serving. Carrot 58 grs, tomato 118 grs, beans 81 grs, peas 42 grs, onion 74 grs per serving.	< or equal 200 g/day of fruits and vegetables
Fish	3 portions per week (trout or tuna). Trout of 80 grs per serving or tuna 120 grs per serving.	< 1 portion per week of any type of fish
Meat	Preferably white meat, beef once a week. Meat 100 grs per serving or chicken 80 grs per serving.	On demand, consumption of any type of meat during the week
Legumes	Two times for week (lentils, beans, chickpeas, blanquillos) 120 grs per serving.	< 2 times per week
Beverages	Fruit juice (blackberry, tree tomato, lulo, sweet and sour guayaba) once a day without sugar or sweeteners of 200 cm^3 per serving.	No restrictions

FAWGT, Diet rich in fruits, avocado, whole grains and trout; UD, Usual diet.

		FAWGT	UD
**Kilocalories**	Kcal	701	717
**Proteins**	%	12.2	16.7
**Carbohydrates**	%	47.9	45.5
**Fat**	%	39.9	37.7
**SFA**	%	9.6	17.9
**MUFA**	%	12.96	11.4
**PUFA**	%	12.71	3.38
**Fibre**	g	16.3	0.7
**β-carotenes**	mcg	846	27.6

Table S2. Composition and caloric distribution of the breakfasts used in the postprandial study. FAWGT, Diet rich in fruits, avocado, whole grains and trout; UD: Usual diet; SFA: Saturated fatty acids; MUFA: Monounsaturated fatty acids; PUFA: Polyunsaturated fatty acids.

	Total n= 44	Women n= 34	Men n= 10
Age (years)	50.8 ± 6.3	50.5 ± 6.5	51.9 ± 5.5
Weight	88.64 ± 2.0	86.95 ± 2.4	94.36 ± 3.6
Insulin (µU/ml)	20.77 ± 2.1	18.60 ± 1.3	28.15 ± 8.1
Glucose (mg/dl)	95.09 ± 1.7	93.91 ± 1.6	99.10 ± 4.8
HOMA-IR	5.03 ± 0.59	4.4 ± 1.9	7.3 ± 7.2
TC (mg/dl)	203.50 ± 5.6	203.39 ± 6.7	203.9 ±10.8
HDL-c (mg/dl)	43.13 ± 1.6	44.14 ± 1.9	39.70 ± 2.1
Non- HDL-c (mg/dl)	160.37 ± 5.8	159.24 ± 7.1	164.20 ± 9.8
LDL-c (mg/dl)	122.21 ± 5.3	121.65 ± 6.3	124.12 ± 10.1
VLDL-c (mg/dl)	38.15 ± 2.7	37.58 ± 2.8	40.07 ± 7.6
TG (mg/dl)	190.75 ± 13.6	187.92 ± 14.0	200.39 ± 38.0
hs-CRP (mg/L)	5.13 ± 0.8	5.82 ± 1.0	2.79 ± 0.4
Waist-hip ratio	0.91 ± 0.01	0.89 ± 0.02	0.97 ± 0.03
Body mass index (Kg/m^2)	35.59 ± 0.6	36.15 ± 0.7	33.71 ± 1.0
Fat (%)	42.28 ± 0.6	43.56 ± 0.5	37.94 ± 1.8
Systolic blood pressure (mmHg)	122.7 ± 2.0	120.95 ± 2.1	128.67 ± 5.1
Diastolic blood pressure (mmHg)	81.09 ± 1.4	79.47 ± 1.4	86.6 ± 2.9
Handgrip strength (Kg)	26.62 ± 1.2	24.55 ± 1.0	33.66 ± 3.1

Table S3. Baseline characteristics of the patients included in the study before any dietary intervention. Data shows the mean ± SE. TC: Total cholesterol; HDL-c; High density lipoproteins; LDL: Low density lipoproteins; VLDL-c; Very low-density lipoproteins; TG: Triglycerides; hs-CRP: C-reactive protein.

	FAWGT		*UD*				
Parameter	***0 h***	***4 h***	***0 h***	***4 h***	***p time***	***p diet***	***Diet vs time***
Glucose	94.38 ± 2.0	86.88 ± 1.1	96.04 ± 2.00	90.26 ± 1.4	0.001	0.056	0.202
Total Cholesterol	201.35 ± 5.6	204.29 ± 5.6	200.43 ± 5.39	203.16 ± 5.7	0.013	0.796	0.957
HDL-c	40.99 ± 1.4	38.43 ± 1.4	41.57 ± 1.60	38.86 ± 1.5	<0.001	0.592	0.870
Non-c HDL-c	160.32 ± 5.5	165.80 ± 5.4	158.98 ± 5.56	164.76 ± 5.8	<0.001	0.766	0.944
LDL-c	124.34 ± 5.3	116.59 ± 5.4	118.82 ± 4.76	105.29 ± 5.0	<0.001	0.028	0.108
PCR	4.97 ± 0.5	4.72 ± 0.4	5.16 ± 0.42	5.05 ± 0.4	0.099	0.450	0.559

Table S4. Postprandial changes in the biochemical parameters after 8 weeks of dietary intervention. a) p<0,05 relative to baseline values in the diet. b) p<0,05 between diets in the same time. FAWGT, Diet rich in fruits, avocado, whole grains and trout. UD, Usual diet. Values represent the mean ± standard error. The analyses correspond to ANOVA for repeated measures where were investigated p time, kinetics of the postprandial response; p diet influence, and p value of the interaction of the two factors (diet *vs* time). When post hoc tests were pertinent, we used multiple comparisons with Bonferroni correction.
